# The Role of Albumin in Human Toxicology of Cobalt: Contribution from a Clinical Case

**DOI:** 10.5402/2011/690620

**Published:** 2010-10-31

**Authors:** Simona Catalani, Roberto Leone, Maria Cristina Rizzetti, Alessandro Padovani, Pietro Apostoli

**Affiliations:** ^1^Section of Occupational Health and Industrial Hygiene, Department of Experimental and Applied Medicine, University of Brescia, P.le Spedali Civili 1, 25123 Brescia, Italy; ^2^Department of Public Health and Community Medicine, University of Verona, 37129 Verona, Italy; ^3^Department of Medical and Surgical Sciences, Unit of Neurology, University of Brescia, 25123 Brescia, Italy

## Abstract

The distribution and adverse effects, especially to optic and acoustic nerves, of cobalt released from a hip arthroplasty and its association with albumin were studied. The analysis of cobalt was performed in plasma, whole blood, urine, and cerebrospinal fluid by inductively coupled plasma mass spectrometry (ICP-MS). The fraction of albumin binding the metal was determined by colorimetric assay using dithiothreitol (DTT). In all the biological matrices very high levels of cobalt were measured, but contrary to expected, a higher concentration in whole blood than in plasma was observed. The determination of altered albumin confirmed this hypothesis. This evidence might indicate an alteration in the binding of cobalt to albumin and a consequent increase in the concentration of the diffusible (free) fraction of the metal. This appears an interesting starting point for further investigations for identifying and better understanding cobalt neurotoxicity, apparently not so frequent in occupational medicine and clinical practice.

## 1. Introduction

Last year we reported the case of a 58-year-old woman with high concentration of cobalt released from her hip arthroplasty. The woman presented a selective II and VIII cranial nerves impairment and mild distal sensory-motor disturbances, and some months later she became definitely blind, severely deaf, and wheel-chair bounded due to severe limbs motor weakness [1].

During surgery of prosthesis removal of a massive infiltration of periprosthesis tissues by metallic debris (metallosis) became evident.

The patient has undergone chelation therapy with ethylenediaminetetraacetic acid (EDTA) in order to reduce the cobalt body burden.

In the scientific literature there are only five other cases of severe adverse effects on nervous system associated with cobalt released from prosthesis [2–6].

Moreover, cobalt neurotoxicity for acoustic and optic nerves has been reported following occupational exposures to the metal [7, 8] or, few decades ago, after oral intake during therapy of refractory anaemia [9–11].

Cobalt concentrations in biological fluids have been investigated in subjects with prosthesis [12–14], but the kinetics of released metal ions is not adequately known, due to the lack of information about amount and rate of release from hip surface, about the solubility of released ions and or about the fraction phagocyted by macrophages and the role of metal deposition-storage mechanisms [15–17].

The distribution of cobalt in the body is also influenced by binding to the plasma proteins. Albumin has been indicated to be a physiological carrier of cobalt ions in old and more recent studies [18, 19].

The cobalt binding to albumin has been studied because the base of the “Albumin Cobalt Binding test” (ACB test) approved by the Food and Drug Administration for evaluating myocardial ischemia.

Ischemia modified albumin (IMA) is a form of human serum albumin in which the N-terminal amino acids have been altered so as to be unable to bind transition metals [20].

However, other pathologies such as trauma, sclerodermia, diabetes, bacterial or viral infections, end-stage renal disease, liver cirrhosis, brain ischemia, peripheral arterial disease, and cancer are able to influence the binding site of cobalt in albumin [21–26]. In addition there are also evidences of possible genetic variants of human serum albumin, particularly structural changes at the N-terminus abolish transition metals binding [27, 28].

The direct measurement of cobalt levels in biological matrices and protein binding of this clinical case may be useful in understanding effects and variability of cobalt neurotoxicity.

## 2. Material and Methods

### 2.1. Cobalt Analysis

Cobalt in blood, plasma, urine, and cephalorachidian fluid was measured using an inductively coupled-plasma mass spectrometry (ICP-MS, ELAN DRC II, Perkin Elmer, Waltham, USA) equipped with Dynamic Cell Reaction. The detailed method is described in a our previous paper [29].

The samples of plasma and blood were diluted with Triton X-100 0.05%, while for urine and cephalorachidian fluid was used bidistilled water, tracepur for inorganic trace analysis (Merck KgaA). The calibration standards were prepared by standard solutions of single elements ranging from 0.5 *μ*g/L to 1000 *μ*g/L cobalt (in HNO_3_ 2% mono elemental standard solution Carlo Erba Reagenti, Milano, Italy).

The accuracy of the method was assessed using specific certified materials for different matrices 1A/B (blood samples), 11A/B (plasma) 2A/B (urine), all from the German Society of Occupational and Environmental Medicine (Erlangen, Germany). The sample solutions were pumped in the spray chamber by a peristaltic pump detecting mass 59(Co).

The accuracy of methods for the determination of cobalt in blood and urine was 95%, whereas it was 97% for plasma. Method precision was 6.1 and 6.9% for blood and plasma determinations, respectively, whereas in the case of urine precision was 4.1%. For each matrix, the LODs were determined as three SD of the background signal. The LOD was 0.02 *μ*g/L for Co–B and Co–P, and 0.05 *μ*g/L for Co–U.

### 2.2. IMA Determination

The concentration of IMA can be determined by addition of known amount of cobalt to serum or plasma specimen and measurement of the unbound cobalt by colorimetric assay [20, 23, 30–32].

To remove the interference of EDTA, the plasma was pretreated with calcium chloride (CaCl_2_ x 2H_2_O; Sigma-Aldrich); the combination of sample and reagent pre-treatment was then centrifuged at 1000–1200 g for 10 minutes without mixing the two components [33].

Plasma was incubated with a cobalt chloride solution (Sigma-Aldrich, CoCl_2_·6H_2_O; 1 g/L, 10 minutes) and dithiothreitol (Sigma-Aldrich; 1.5 g/L, 2 minutes) before dilution in saline solution (0.9% NaCl) and measurement at 470 nm in a spectrophotometer (UV-Vis spectrophotometer Cary 50, Varian, Palo Alto, USA). IMA was calculated from the difference between samples measured with and without dithiothreitol and reported in mean absorbance units (ABSU). Bar-Or et al. [31] showed that values greater than 0.400 ABSU indicate lower cobalt binding and values equal or lower than 0.400 ABSU higher cobalt binding.

### 2.3. Statistical Analysis

Data were analyzed by the SPSS 16.0 for Windows statistical package.

The distributions, assessed by the one-sample Kolmogorov-Smirnov test, were normal for plasmatic and hematic cobalt in controls and for IMA in both groups.

The trend of cobalt distribution in woman's plasma and blood and in CoB/CoP ratio was assessed by Ordinary Least Square (OLS).

The range of repeated measures of cobalt concentrations, ratio CoB/CoP and IMA in woman with prosthesis were compared with the arithmetic means of healthy and unexposed subjects.

## 3. Results and Discussion

**Figure 1:**
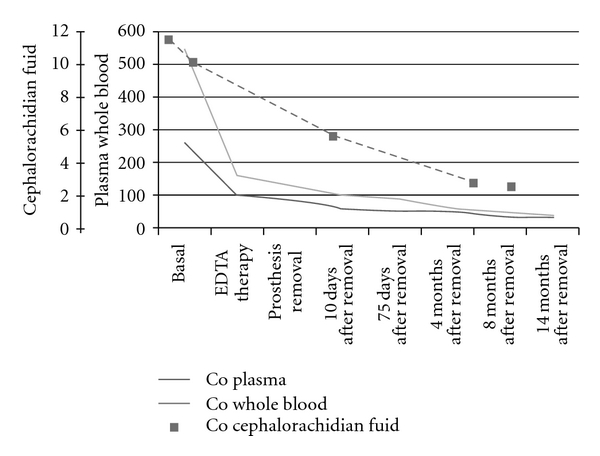
Levels of cobalt (*μ*g/L) in plasma (CoP), whole blood (CoB), and cephalorachidian fluid (CoL) in woman with prosthesis. The normal values are CoP 0.1–0.6 *μ*g/L; CoB 0.05–2.7 *μ*g/L; CoL 0.05–0.15 *μ*g/L.

**Figure 2:**
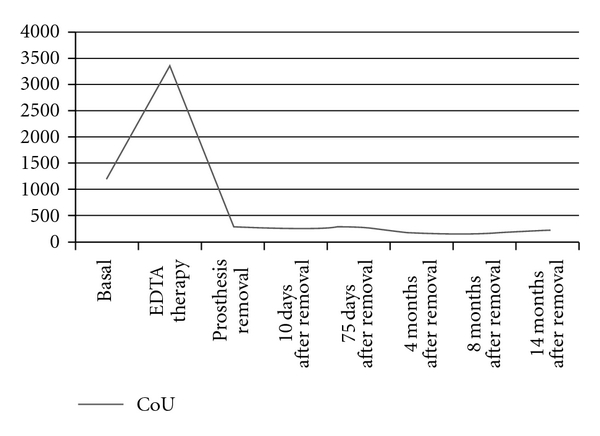
Levels of cobalt in urine (CoU) in *μ*g/L in woman with prosthesis. The normal values are 0.1–1.5 *μ*g/L.

Trend of plasma, whole blood, and urine cobalt concentrations are reported in Figures 1 and 2. Cobalt basal levels were very high (549 *μ*g/L in whole blood; 260 *μ*g/L in plasma), after the chelation therapy the levels decreased by 70% in whole blood and 61% in plasma. A further reduction occurs after the prosthesis removal (94 *μ*g/L in whole blood and 64 *μ*g/L in plasma).

The current (14 months after removal) cobalt levels remained higher than reference values (33.9 versus 0.05–2.7 *μ*g/L in blood; 28.5 versus 0.1–0.6 *μ*g/L in plasma).

In the urine cobalt was rapidly and in great extent eliminated after a chelation therapy, the rate of excretion was 2173 *μ*g/L and stabilized around 200 *μ*g/L after prosthesis removal.

The cobalt in the cephalorachidian fluid was determined before chelation and removal of the prosthesis (11.4 *μ*g/L and 10.1 *μ*g/L) and two days after (5.4 and 2.6 *μ*g/L); the normal values ranged between 0.05–0.15 *μ*g/L.

In spite of these high levels a clear improvement of clinical evidence for most adverse effects to nervous system, except the visual function, was seen.

The investigation of cobalt distribution in blood fractions showed, contrary to the expected, that most of the cobalt was in whole blood than in plasma.

Normally most of the cobalt is found in the serum and plasma with lower concentrations in the red blood cells (RBC); the whole blood levels are between those of the RBCs and the plasmatic fraction [34].

The ratio CoB/CoP is different when considering different kind of metal exposures, in Table 1 were reported the ratio of workers exposed to cobalt during hard metals production (unpublished data) and a collection of samples from patients after hip arthroplasty [34].

A greater distribution of cobalt in whole blood than in plasma may indicate an alteration in the binding of cobalt to plasma proteins and an increased concentration of nonprotein-bound cobalt.

**Figure 3:**
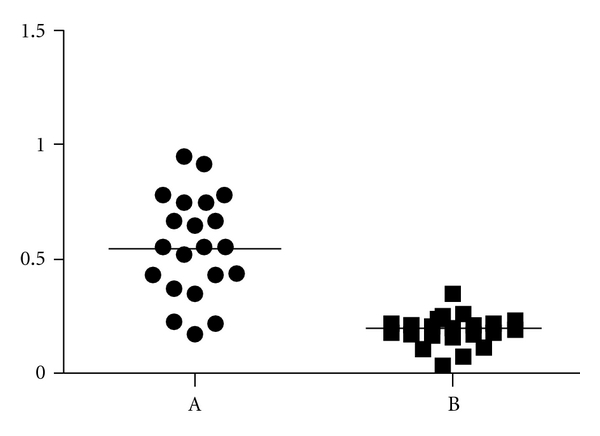
Cobalt-HSA binding absorbance (ABSU) in the woman with prosthesis (repeated measures on different days) (A) and in healthy unexposed subjects (B) (mean).

IMA was measured in different samples of patients in study and in healthy unexposed subjects, IMA values significantly higher than controls seem to confirm this hypothesis (Figure 3).

According to Bar-Or [31] the absorbance units (ABSU) greater than 0.4 indicate a reduced cobalt binding to albumin.

**Figure 4:**
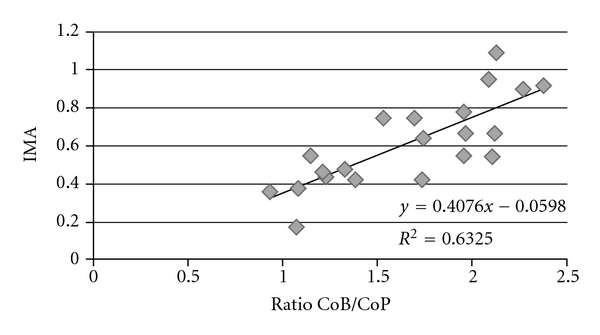
Correlation between ratio blood cobalt (CoB)/plasma cobalt (CoP) and ischemia modified albumin (IMA), expressed in absorbance units.

IMA's values and ratio of CoB/CoP showed a good correlation (*r* ^2^ = 0.6325) (Figure 4); on the contrary, no correlation has been demonstrated between the values of IMA and the concentrations of cobalt in blood or in plasma.

Cobalt in blood and plasma follows a defined decreasing trend while the ratio of CoB/CoP does not follow any specific trend.

Critical points of this hypothesis have been considered; the possibility that in our conditions the albumin binding site could be only saturated by high amount of cobalt and not altered is disproved by the fact that altered albumin (IMA 0.92 and 1.17 ABSU) corresponds both to high and lower cobalt blood concentrations (415 and 38 *μ*g/L, resp.), excluding a possible interference of cobalt concentration on colorimetric measure.

The percentage of blood albumin on total proteins was monitored during the hospitalization period and was 55.68 ± 9.57% (normal range of laboratory 55–69%).

The alteration of N-terminal binding site of human albumin may be the cause of the reversed CoB/CoP ratio and the consequent greater amount of cobalt in an available form for higher distribution and interaction into the cells.

Nonprotein-bound metal can enter cells through a promiscuous metal transporter, divalent metal transporter 1 (DMT1) [35, 36], that is known to actively transport iron, zinc, manganese, cobalt, cadmium, copper, and lead, via a proton-coupled mechanism [37]. DMT1 is predicted to have 12 transmembrane segments and to be expressed in neurons, allowing the incorporation of metals from the extracellular environment and/or from the recycling endosomes.

Howitt et al. [38] showed that human neurons in culture were susceptible to metal toxicity in cellular assays involving increasing concentrations of environmental iron and cobalt. They have identified DMT1 as the key player in the entry of metal ions in the brain.

In our case an high concentration of free cobalt in the whole blood could be available to interact with DMT1 and becoming the cause of the neurological effects.

The concentration of cobalt in cerebrospinal fluid measured before chelation and before prosthesis removal showed a massive amount of cobalt (11.4 *μ*g/L), that corresponds to a 2.37 CoB/CoP ratio.

An alteration of the bound with the main plasma protein resulting in a greater amount of free cobalt may be the cause of the not frequently neurologic symptoms reported in the scientific literature for cobalt intoxication.

Some authors suggest that the alteration of binding site of albumin could be the result of acidosis, reduced oxygen tension, and the generation of free radicals [39, 40] and this conditions could be caused by cobalt and chromium constantly released from the prosthesis.

In addition genetic variants of human serum albumin have been reported, each of these variants has been classified on the basis of the structural changes at the N-terminus that abolish transition metal binding [41].

Another aspect to consider is the possible influence of diabetes. Our patient implanted with hip prosthesis was suffering from type 2 diabetes.

Many authors reported higher levels of IMA in diabetic patients, and they suggest that the plasmatic albumin of diabetic patients was modified for the chronic hypoxia conditions provoked mainly by hyperglycemia and oxidative stress in diabetes [25, 41].

Ukinc et al. [42] reported that diabetic patients without cardiovascular disease have a higher serum IMA levels compared to healthy controls.

Diabetes may have been a contributory cause of the alteration of albumin that in the state of cobalt intoxication was not able to correctly bound it favoring the increase in the haematic fraction.

## 4. Conclusion

Among all the patients with extremely high cobalt concentrations in blood or urine, only few develop a severe neurologic syndrome. The reason is still unclear and, even if it is certain that cobalt can cause potential serious effects on human central nervous system, little is known about the mechanisms through which this metal can cause neurotoxicity to optic and acoustic nerves.

We have tried to explain the anomalous reaction to cobalt in different ways and an alteration of plasma protein with a consequent more active fraction seems to be the most plausible hypothesis for the neurological symptoms.

A possible explanation for the symptoms may be the altered albumin binding capacity and the consequent increase of the free form of cobalt in the blood.

The decreased linkage to albumin for cobalt generates IMA and increases the concentration of biologically active free cobalt. The free cobalt can then interact with specific protein carriers to the cellular targets of the central nervous system.

The cause of albumin alteration is not clear, we hypothesize an influence of several factors, which in this specific situation are the generation of free radicals by cobalt, a genetic predisposition or a possible influence of diabetes.

We hope that our study can be the starting point for further studies on the neurological effects related to the toxicity of cobalt.

## Figures and Tables

**Table 1:** Ratio of whole blood cobalt (CoB)/plasma cobalt (CoP) in present study (repeated measures), workers exposed to cobalt, and ratio reported in a study of Walter et al. [34]. mean ± standard deviation.

	N°	CoB/CoP	CoB (*μ*g/L)	CoP (*μ*g/L)
Present study	61	1.73 ± 0.38	196.12 ± 134.45	106.36 ± 57.49
Workers exposed to cobalt	23	0.90 ± 0.40	3.20 ± 1.52	3.86 ± 1.84
Walter et al., [34]	29	0.82		
